# ‘There’s a will, but not a way’: Norwegian GPs’ experiences of collaboration with child welfare services – a grounded theory study

**DOI:** 10.1186/s12875-024-02269-9

**Published:** 2024-01-24

**Authors:** Oda Martine Steinsdatter Øverhaug, Johanna Laue, Svein Arild Vis, Mette Bech Risør

**Affiliations:** 1 Department of Community Medicine, The General Practice Research Unit, UiT The Arctic University of Norway, Tromsø, Norway; 2 Department of Community Medicine, UiT The Arctic University of Norway, Tromsø, Norway; 3 Regional Centre for Child and Youth Mental Health & Child Welfare, UiT, The Arctic University of Norway, Tromsø, Norway; 4 The Research Unit for General Practice and Section of General Practice, Department of Public Health, University of Copenhagen, Copenhagen, Denmark

**Keywords:** General practice, Child welfare services, Child protective services, Cross-sectoral collaboration, Vulnerability, Child abuse and neglect, Child maltreatment

## Abstract

**Background:**

Adverse childhood experiences can have immediate effects on a child’s wellbeing and health and may also result in disorders and illness in adult life. General practitioners are in a good position to identify and support vulnerable children and parents and to collaborate with other agencies such as child welfare services. There is a need for better integration of relevant services. The aim of this study is to explore GPs’ experiences of the collaboration process with child welfare services.

**Method:**

This is a qualitative grounded theory study, with data consisting of ten semi-structured interviews with general practitioners across Norway.

**Results:**

The doctors’ main concern was: ‘There’s a will, but not a way’. Three subordinate stages of the collaboration process were identified: (I) Familiar territory, with a whole-person approach to care by the doctor. (II) Unfamiliar territory, when child welfare becomes involved. Here, a one-way window of information and a closed door to dialogue perpetuate the doctors’ lack of knowledge about child welfare services and uncertainty about what is happening to their patients. (III) Fragmented territory, where doctors experience lost opportunities to help and missing pieces in the patient’s history.

**Conclusion:**

General practitioners are willing to contribute to a collaborative process with child welfare, but this is hampered by factors such as poor information flow and opportunities for dialogue, and limited knowledge of the partner. This implies lost opportunities for doctors to help families and contribute their knowledge and potential actions to a child welfare case. It can also impede whole-person care and lead to fragmentation of patient pathways. To counteract this, electronic two-way communication could enable a collaborative process and relationships that enhance coordination between the parties. Making space for all parties and their individual roles was considered important to create a positive collaborative environment.

**Supplementary Information:**

The online version contains supplementary material available at 10.1186/s12875-024-02269-9.

## Background

Several mental and physical health problems frequently seen in family/general practice have been linked to negative childhood experiences. Adverse childhood experiences can have immediate effects on a child’s wellbeing and health and may result in disorders and illness in adult life. Improving the childhood environment can help to prevent development of health problems. The goal of the Norwegian child welfare services (CWS) is to support children and adolescents living in conditions that represent a risk to their health or development.

As early as 1998, Felitti et al. established a strong dose–effect relationship between the number of adverse childhood experiences of children and the disease burden they developed later in life [1]. They linked these experiences to the development of mental health problems such as substance abuse and depression, but also to physical diseases such as heart disease, chronic obstructive pulmonary disease and cancer. Several studies have replicated and expanded on these results [2–4]. On a global scale, child abuse and neglect have been estimated to affect every second child [5]. A Norwegian study from 2019 explored the prevalence of child abuse and neglect among Norwegian youth and found that 20 percent had experienced physical violence by a parent [6]. Five percent had been subjected to severe types of physical violence, such as being beaten, and a similar percentage had experienced psychological violence, such as repeated humiliation and threatening. In many cases, a general practitioner (GP) will have some knowledge of families at risk, because this often coincides with a poor parental state of health. Strong risk factors included socioeconomic status, parental substance abuse and mental illness [6]. 

GPs are in a unique position to uncover and respond to child abuse [7] and Norwegian GPs consider themselves to be in a good position to discuss children’s situation in consultations with the parents, when a parent has a medical condition that might affect their caring ability [8]. Most studies focus on how GPs or primary care workers recognize vulnerability in children and their reporting practice to the CWS [9–11], and they show that GPs generally underidentify and have a lower reporting rate to the CWS than expected [12–14]. The barriers identified as preventing GPs from contacting CWS are among others structural obstacles such as time pressure and confidentiality, fear of affecting the patient-doctor relationship, negative prejudices against CWS and lack of knowledge about risk factors [10, 15, 16]. A need for better collaboration between general practice and CWS has been indicated several times [11, 14, 16–21] and there is a lack of intersectoral communication between the health care sector and child welfare sector [14]. We have not found research that focus directly on a collaborative process between GPs and CWS, although studies have been conducted on GPs’ collaboration with other agencies in the health care sector, such as nurses, pharmacists, mental health workers, physiotherapists and social workers working within the health care sector. They point out several barriers to collaboration, including shared information and confidentiality [22, 23]. A scoping review has been conducted on collaboration between GPs and social workers that indicated benefits on behalf of patients, professionals and healthcare systems, but studies concerning children were not included [24].There are theories on the concept of inter-professional collaboration [25, 26]. This has been defined as a type of relation and interaction where different forms of sharing (e.g. shared responsibility, shared decision-making, shared values or shared data) is used as collective action towards a common goal. The theory of relational coordination proposes that coordinated collective action is best achieved through a relationship of shared goals, shared knowledge, and mutual respect, supported by frequent, timely, accurate, and problem-solving communication [27]. Some of these aspects may also apply to the collaboration between GP and CWS, in particular shared values and shared data. Shared decision-making on the other hand is not commonly seen as a key feature of this relationship. It is also not clear to which degree GPs and CWS have shared responsibilities beyond the very general principle of the child’s best interest. For example, the GP may have responsibilities towards parents as patients that are not shared with CWS. Therefore, we think it is important to understand the involved actors’ perspectives on and experiences with the respective partner. 

Despite empirical and theoretical knowledge about collaboration processes in general and between general practitioners and public services specifically, the conditions, challenges, and consequences for a collaboration process between GPs and CWS is not yet understood. With this study we aim to explore GPs’ experiences of the collaboration process with CWS. A grounded theory approach is therefore considered appropriate.

## Methods

### Study design

We conducted a qualitative grounded theory study to explore GPs’ experiences of collaboration with the CWS, with an analysis based on Corbin and Strauss [28]. Grounded theory is a well suited method for exploration of a phenomena, subjective experience, social processes and interactions [28] and where existing models have been developed but not tested on the respective study sample [29].

The data corpus consists of semi-structured interviews with GPs. This approach was chosen because GPs often work and make decisions alone. Their individual opinions and experiences are fundamental to their actions and decisions on collaboration with CWS. The research team had an open approach to the research questions, and no hypothesis was established for the study, in accordance with the methodology. However, the first author’s experience in the research field from working as a GP, gave rise to the motivation for the study with an assumption that the collaboration between GPs and CWS had room for improvement. This paper follows the Consolidated Criteria for Reporting qualitative Research (COREQ) [30] (see Supplementary file 1).

### Context/setting

In Norway, general practice is the patient’s first point of contact with many health services. Citizens have a right to belong on a GP’s list, and most contacts with health care go through that specific GP. The GPs have a key role in coordinating care with other health and social services on behalf of their patients. The GPs’ duty of confidentiality is defined in the Health Personnel Act, which allows for exceptions in cases regarding factors that could harm a child, and Norway has mandated reporting for all professionals when there are concerns about child maltreatment [31]. The threshold for reporting is generally low and most reports are investigated by the Norwegian CWS [32]. When the CWS decides to open an investigation, they may ask for parental consent to collect information and cooperate with other parties such as the GP. If such consent is given there are no legal restrictions for the exchange of confidential information between the parties. If there is no parental consent the CWS can only require information from a GP if the case is serious enough to warrant a court petition for child removal or mandatory medical treatment [31]. The CWS is decentralized to the municipalities, but services can be intermunicipal in rural areas, or organized in local districts in cities. Larger CWS often have specialized teams, while employees in smaller CWS work as generalists.

### Participants

We purposefully sampled GPs from urban and rural areas in Norway, with at least five years of experience as a GP to increase the chances of their involvement with CWS cases. We used our professional extended network to reach possible informants from locations that fitted the geographical profile. The first author (OMØ) contacted suitable GPs via e-mail. Four of the informants were partly known to the first author, through an extended professional network. We contacted 14 GPs during our sampling period. One did not reply, and one declined the invitation based on her work experience. Thus, 12 GPs agreed to be interviewed, but two of these did not have time during the data collection period. Of the ten informants, seven were female, and three were men. They were aged from 40 to 70 years old, most of them being in their forties. One worked in the biggest city in Norway (over 700 000 inhabitants), two in bigger cities (between 70 000 and 90 000 inhabitants), two worked in medium-sized towns (between 20 000 and 50 000 inhabitants), and five in small towns and villages in rural areas (between 2000 and 8000 inhabitants).

### Data collection

The data were gathered from February 2020 to April 2021. OMØ conducted all ten interviews; six took place in the GP’s surgery or a nearby suitable location and four were held online. No other participants than the participating GP and the interviewer were present during the interview. All the data were recorded, and the duration was approximately an hour. Field notes were not made during the interview session, but reflective notes were made after the interview was conducted. Eight interviews were transcribed and anonymized by OMØ, and two by an external party. The transcripts were not presented to the participants for comments. The first author worked as a GP and a part time PhD-student at the time of the interviews. As part of her PhD-studies she received training in qualitative research methodology and interview techniques. The interviews were based on a study-specific interview guide with stimulus material, developed by OMØ and MBR (see Supplementary file 2). The pilot interview provided rich data and was thus included in the data corpus. The interview guide underwent several minor revisions in response to the initial coding and category identification during the ongoing parallel analysis. These changes were especially relevant in the beginning of the data gathering, in order to adjust the interview guide to the theoretical sampling, and at the end, to reach theoretical saturation of our core concepts [28]. When this was achieved, we stopped collecting data. No repeated interviews were conducted. The participants were orally informed about the interviewer’s background and motivation for the study at the beginning of the interview. The consent form also included written information about the background of the study.

### Data analysis

**Fig. 1:**
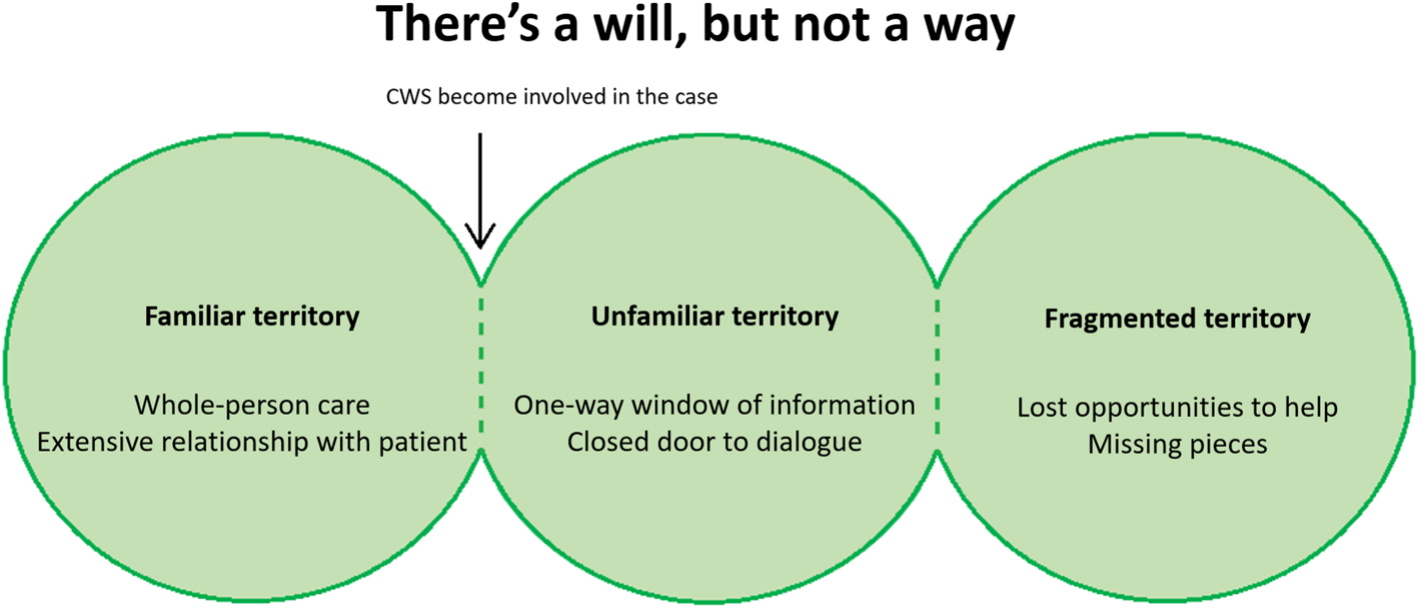
A model of GPs’ experiences of collaboration with CWS. This is a conceptual model describing the GPs’ main concerns regarding collaboration with the CWS, based on the concept of *There’s a will, but not a way*. The figure illustrates shifting stages of GPs’ experience with their patients in situations where the CWS become involved. The first stage describes the GPs’ *familiar territory*, providing health care to their patients, with an emphasis on *whole-person care* and often consisting of a long and *extensive relationship* with a patient. In the first stage, the CWS is not part of the care, but when they become involved with a patient or family on the GP’s list, the conditions change to the second stage. This is characterized as *unfamiliar territory* by the GP. Obstacles such as a *one-way window of information flow* and a *closed door to dialogue* help to perpetuate the GPs’ lack of knowledge of the CWS’ procedures and work methods, as well as direct information on what is happening to their patients. This leads to *fragmented territory*, affecting GPs’ possibilities to do their job, expressed as *lost opportunities to help* and *missing pieces* in the patient’s history

In accordance with the grounded theory method, the analysis runs parallel to data collection [28]. (see supplementary material for figure of analytical process). After having conducted the first interview, OMØ wrote analytical notes (memos) and identified, labelled and coded segments of meaningful raw data (open coding) [28]. NVivo 12 [33]. was used for some of the initial/open coding, but not for the later analytical process. The data corpus and initial codes were discussed with MBR. Partly parallel to this process we discussed how already identified codes could be related and categorized (axial coding) and all four authors participated in this of selected parts of the corpus. Analytical strategies included analysis of similar issues and situations from different parts of the data corpus in order to differentiate and nuance the emerging categories (constant comparative analysis) [28, 34]. and examining and elaborating on an issue’s or situation’s meaning by relating it to existing literature or experience (theoretical comparative analysis) [28]. In the final step, we identified and developed core concepts and integrated them into a model (Fig. 1) (selective coding and theoretical integration). The participants have not been asked to provide feedback on the findings.

## Results

### ‘There’s a will, but not a way’

In this study the GPs’ main concern about their collaboration with the CWS was that they had a will to contribute, but a feeling that there was no way to do so. The main theme is illustrated through the model seen in Fig. 1. The GPs described that they missed opportunities to contribute in CWS cases, in the interest of the patient, the CWS and themselves as GPs. The problem was partly due to systemic factors such as a lack of effective (electronic) communication, what they felt to be different levels of confidentiality and a general lack of knowledge from the GPs themselves about CWS’ work and function. Some GPs had stopped getting involved in these cases, even though they believed that cooperation with CWS would benefit all parties involved, because they were not being provided with sufficient information and had a feeling of being left out.

I feel that they definitely don’t want me as a team player in supporting vulnerable children and families. (GP4).

### Familiar territory

#### An extensive relationship with the patient

The first stage of the model represents the daily practice of the GPs with their patients. All GPs in this study emphasized that they usually had a long and/or *extensive relationship* with their patients, over a long period of time and including much information from different sources. The GPs were often involved in a variety of situations with their patients, collaborating with different agencies.

I’ve known these people for over thirty years, and the ones who come now with little children, I’ve seen them myself at the infant health centre. So you have a long-term perspective on how families are knit together and function. (GP5)

Notably, when using the word patient(s), the GPs in this study often referred to the parent on their list or the family as a whole. They seldom referred to the child alone. The GPs talked generally about their experiences, but most of the examples they used referred to highly complex situations of cases involving CWS.

GPs occasionally received a letter from the CWS about a family they hardly knew, but in many cases the patients who became involved with the CWS already had a history of contact with their GP or other health care services. This meant that the GP was familiar with the patient’s history and situation. The GPs experienced that their knowledge of the patient would be useful for the CWS case, such as contributing a piece to the jigsaw puzzle and expanding the perception of the patient.

I found that the CWS came in with a very stereotypical and moralizing approach. And then I told them that this mother had a very, very strong will to get by. She had a lot of will to cope, relatively good abilities, so there were a lot of strengths to use (…) her mother was a resource, and there were friends that I knew of around her (…) and after a while the CWS was able to activate those resources. (…) This is just an example of one of the times I’ve found that my knowledge of the patient from several years back can be of use, to the CWS as well. (GP3).

#### Whole-person care

The GPs in the study highlighted their patient-centred approach, with examples that may theoretically be understood as *whole- person care*, holistic patient care or a biopsychosocial approach [35]. They emphasized that information about various aspects of the patient was important for them as GPs, such as the patient’s psychological, social and cultural background. This information complemented the biomedical understanding of the situation and the symptoms of the patient. GPs had the power to act on some of these aspects, while others were helpful in understanding the complexity of the situation.

I think it’s always important to explore all the sides of a situation to get the whole picture. It’s no use just asking: Are you having any side effects from your medication? You must have a—we must always bring the patient’s whole life into the consultation, in fact. (GP10).

In complex cases, the patient/family often had a combination of conditions that influenced each other, such as medical conditions (mental and/or physical), social vulnerability and cultural challenges. The GPs described that being in good health meant more than not having a disease. Hence, they often saw the need to cooperate with other agencies outside their surgery to contribute to a health promotion process.

You’re trying to form a ring around that family to help it function well - my idea is that you have to make an arrangement around them, which naturally involves others [other agencies]. (GP2)

I think it could be useful to collaborate more. (…) I think there’s a risk of silo thinking. We think about our things, and the child and youth mental health services think about their things, and the CWS about their things. And then it’s difficult to have a holistic approach to these patients. (GP9)

The GPs were used to linking up different systems around individual patients or families, involving several agencies, usually in primary care, such as home nursing or primary mental health care. This whole-person approach gave the GPs a powerful tool to do their job in complex cases. They saw this as one of the pillars of their profession as GPs, and part of their responsibility to their patients.

The core of GPs’ work with vulnerable patients and families were optimally an *extensive relationship* with the patient, and an integration of knowledge from that relationship into *whole-person care*. Even though cases could be difficult and complex, this was perceived as comfortable and *familiar territory* to GPs.

### Unfamiliar territory

GPs in this study described that they had little knowledge of CWS’ procedures, work methods and how they evaluated cases. This led to *unfamiliar territory* when CWS became involved with a patient.

#### A one-way window of information

The *unfamiliar territory* described by the GPs is maintained by a limited information flow between them and the CWS. This is conceptualized as a *one-way window of information*. Unlike the feedback the GPs were used to receiving from partners, such as discharge summaries from hospitals, they felt they did not receive any response to their assessments in a CWS case. This lack of feedback made it difficult for them to learn from the cases and improve their ways of contributing. Moreover, rarely receiving details of what had happened in a case resulted in a general lack of knowledge about the procedures and work methods of the CWS.

Interviewer: Can you think of anything else that makes collaboration more difficult? GP: Well, it’s the thing that you don’t get any – you get very little – you sit there like a satellite, and then you give information and you never get any feedback. (GP6)

The GPs were frustrated with a feeling of having to give everything but not getting anything back. This was perceived as a huge obstacle to collaboration, with various consequences, such as limited opportunities for the GP to help the patient and the case.

#### A closed door to dialogue

In addition to the *one-way window of information*, the GPs felt that they met a *closed door to dialogue*, resulting in a feeling of secrecy and difficult collaboration.

I feel it’s like a closed door, with a lot of secret things going on behind it (…) But of course, those are personally sensitive things, so it might be okay that that door isn’t – that door is – you don’t really know what’s happening behind it. (GP1)

They understood and respected the fact that the CWS worked with personally sensitive material, and that confidentiality was important. Several of them had no interaction with the CWS caseworker at all, except for a possible letter with a request for information. The main reason why the CWS did not exchange information and did not engage in a collaborative dialogue as much as the GPs would have liked was thought to be confidentiality legislation.

I think there’s legislation behind it, that’s the reason why they can’t have two-way communication. (GP4)

The GPs did not perceive their confidentiality as an obstacle to potential collaboration. Furthermore, having consent from the patient meant that they could collaborate more freely. The GPs assumed that the CWS had a stronger duty of confidentiality than they had themselves.

*The closed door* included systemic factors such as the lack of an open, simple channel of communication and the possibility of an ongoing mutual dialogue when working simultaneously with a patient or a family. Collaborations that worked well for the GPs were easy and effective access to means of communication for both parties, optimally via electronic channels or in some situations with a phone call, where it was easy to reach the right people. This contributed to the general feeling of *familiar territory*, where the GPs had sufficient knowledge of and communication tools to reach their partners.

I think having the possibility to send digital messages is a huge advantage. It’s a part of the collaboration that’s important, and it makes it quicker to communicate. Thinking about calling each other, when everyone – well, that doesn’t work well at all. When I have patients all day, and the others are often in meetings, and when I have time to phone them they’ve mostly gone home for the day. (GP8)

Between the healthcare sector and the CWS there were no possibilities for electronic messages that corresponded to the GPs’ electronic patient record systems. The possible contact methods were by letter, phone calls or physical meetings. All GPs mentioned that it would improve dialogue if the CWS could be reached with electronic messages satisfying confidentiality standards, which they used with other partners, such as the hospitals, social services and other primary care services. They saw this as more effective and easier to fit into a busy schedule, and messages regarding collaboration were often sent and replied to outside office hours.

The GPs described a *one-way window* of information due to a lack of useful information in return from the CWS, and a *closed door to dialogue* which limited the opportunities to work together in the best interests of patients and families.

### Fragmented territory

Limited information flow and options for dialogue with CWS led to difficulties with fragmented pieces of information, described in the last stage of the model (Fig. 1). The GPs described that this had negative influence on their ability to provide optimal help to the case and care to the patient.

#### Lost opportunities to help

An obstacle expressed by the GPs was their lack of opportunity to provide relevant information to the CWS. During an ongoing CWS investigation, GPs felt they ideally could help by providing useful patient information. With patient consent GPs are not bound by their duty of confidentiality, but the information they provide should still be considered necessary for the purpose. When the CWS has reason to believe that a child is abused, neglected or has serious behavioural difficulties, GPs are obliged by law to give information that is requested from the CWS, irrespective of the patient’s consent. The CWS makes a legal decision about this, but GPs are not told the reasons for the decision. Such requests often include specific questions that GPs need to answer. However, not knowing the background to the case can make it difficult to consider what exactly is relevant information necessary for the purpose. One result is that GPs may find that they are obliged to reveal unnecessary aspects of patients’ privacy.

I sometimes think the law should be changed, so the CWS have the chance to tell a doctor a bit more about why they need information. I’ve found, in all cases, that they ask about things that are very private, and I can’t understand what they’re going to do with them. Sometimes it seems to me to border on personal curiosity. (GP3)

Little knowledge about the general procedures in the CWS adds to this concern about revealing unnecessary details. It might also add to a mistrust from the GPs towards the CWS, thus misinterpreting genuine requests as personal curiosity. This can make GPs wary about what patient information they should provide, as they do not know how it will be used. Another consequence is that relevant information and assessments a doctor has made will be unknown to the CWS if not specifically requested. Medical records are not written on the basis that they might be used in a CWS case.

Interviewer: So if you have, as you say, some observations that you’ve made in the back of your head because you know the patient, but you haven’t put them in the record, can you still convey this? GP: Yes, there is a ‘Do you have any other relevant information?’ section, but that’s extremely difficult to complete, because I don’t know what’s relevant. (GP10)

Even though the requests often included the possibility for the GPs to write other relevant information, knowing what information was relevant for the CWS, but without knowing the basis for the request, was perceived as a very difficult task, since GPs have to choose information from years of medical records and familiarity with patients and their environment.

The *fragmented territory* made it difficult for the GPs to provide appropriate courses of action for their patients and entails a poor opportunity to communicate relevant information. The piecemeal information the GPs received from an ongoing CWS case affected their ability to support families and vulnerable children in complex life situations. The GPs in this study had assumed that they would receive an information request every time the CWS had a case involving one of their patients, and all were surprised to learn that the CWS only requests information from the GP in a limited number of cases.

Sometimes I think we could, you know – that it’s fruitful to collaborate a bit more. To exchange information and stuff. (…) There was a boy that had been in the system for many years (…) and at one point there was a note of concern about the care from the father earlier on (…) the father has been here and brought up his anger issues with his GP, and no one has asked about the children for instance. And the same GP has followed up the boy for his behavioural difficulties and challenges, but I don’t think the GP even knew they were related. And then it’s like – the child and youth mental health services and the educational-psychological services have been involved a lot and nobody’s figured out what’s been wrong with that boy. And then active violence has been going on for many years. (GP9)

This GP mentioned the importance of having a complete picture of what is going on around children and in families in order to provide optimal support.

Others talked about their *lost opportunities to help* with different support measures such as sick leave notes for the parents, help in contacting the social benefit system, psychological support or alternative treatments for mental, physical and drug-related illness, which could help reduce the total amount of toxic stress in a vulnerable family. However, it was again difficult to provide this care and support when they knew nothing or very little about the involvement of the CWS. The GPs gave examples of situations where they had used their professional expertise in a positive way for both the patient and the CWS. One example was a debriefing session with a patient following what they considered a difficult experience with the CWS to improve future collaboration. Further examples were to be a neutral third party in difficult meetings with the patient and the CWS, or to provide psychological support for a parent after difficult news from the CWS.

I don’t think in any way that I can solve the problems they have, but at least I can contribute in such a way that collaboration in the other agencies works better. (GP4)

GPs perceived it as meaningful work to speak positively about the CWS to the patient and help prepare the patient mentally for the process, but these examples were atypical of their general experience. In most cases the GPs viewed the scarce information and dialogue as *lost opportunities to help*.

Another GP stated that the barriers to collaboration were structural but had found a way to overcome them:

I think, well, the fact they keep their cards so close to their chest is a structural obstacle with a negative effect on collaboration. But after I’ve become more familiar with them, it’s easier to phone them and get some information after all, without it having to be so official, because we both think pragmatically and see that it’s more beneficial for the children involved. (GP8)

Only this GP and one other described their collaboration with the CWS as good. They had both developed a friendly relationship with CWS staff and were able to establish personal contact that helped them to obtain some of the relevant information they needed to do their job properly. They were also more confident than the other GPs of obtaining unsolicited information about health concerns that the CWS might have.

#### Missing pieces

The *unknown territory* affected the GPs’ in two ways: their ability to assist during a case, and in *missing pieces* in their knowledge about the patient’s background and thus their *whole- person* approach to care. The GPs explained how they took the patient’s lived experiences, diagnoses and environment into account to make sense of their patients’ challenges, and how best to help them and their family. However, *missing pieces* made it difficult for the GPs to assess a situation correctly. One GP drew a parallel to assessments of physical health and explained how knowing about a vulnerability in a family would affect the assessment of the kind of help to be offered.

Just like I’d want to know if you’re in active cancer treatment before I assess your pneumonia. It’s a bit of the same thing. That father for instance, I would offer him a sick leave note quite early on, because I know that when he gets exhausted (…) I know that the family will function poorly. (GP4)

The GPs perceived the different family members as pieces of the same system. They felt that by helping one, they could help ease the total situation for the family, and thus the children.

No, I’ve never got any feedback from the CWS, so I have to ask the patient in order to provide support. That’s interesting because it can absolutely – well, it’s closely linked to the treatment of the patient. If you want to see the patient in a holistic perspective (…) They have very poor finances, and a mother with all her health issues and challenges who’s responsible for the everyday care of the kids at home. So, to ensure that they get good enough everyday care, it’s very important to prevent her going into a new serious depression or serious deterioration of her PTSD, or whatever diagnosis she’s been given. And that’s why it’s a bit strange that we’re not getting any feedback on these things, because it’s all about working holistically with these patients. (GP9)

In addition to complicating the assessment of a situation, *missing pieces* in the patient story made it difficult for the GPs to triage which patients needed most of their attention. Several of them gave examples of how they would give priority to a patient they knew was in a vulnerable position. When they lacked information about such a stressful process as a CWS investigation, or important information of concern about the family, the patients involved were not prioritized as highly as they would have been otherwise.

Interviewer: Would a CWS case affect your work with your patients? GP: Yes, it would, and I think it should. Not to make me more sceptical, but that – well, the relationship aspect is very important for people’s health, both the children and the parents, so a CWS case is very important information to include in a holistic assessment. So I think it affects me, and I think it should. It affects me mainly in making me more attentive to different things. Not observant, in order to report them, but in a way of being more alert if I think that someone needs a bit more than I would have given otherwise. (GP8)

The GPs perceived this lack of information as a *missing piece* in the patient’s story, which made it difficult to provide optimal care.

## Discussion

In this study we found that the GPs had a strong will to engage and collaborate in cases involving vulnerable children and families, based on an approach of *whole-person care* for their patients. GPs believed that they had knowledge and information that would benefit the case, and they had a desire to obtain further information that they felt could enhance their understanding of their patients. The work methods and procedures of the CWS were *unfamiliar territory* for most GPs, which made collaboration difficult. Most GPs described that they received very limited or no information from the CWS, almost like a *one-way window* of information exchange, where they had to give, but did not receive anything back. The door to dialogue was perceived as closed; this was related both to a perception of different levels of confidentiality and to structural hindrances such as the lack of effective electronic communication. These obstacles to collaboration and engagement in cases of vulnerable children and families led to *fragmented territory*. They made it difficult for the GPs to help and contribute for the benefit of the vulnerable families and made it challenging to follow up the patient afterwards with the same *whole-person* engagement.

### Familiar territory: basic care values of a GP as conditions for collaboration

The GPs in our study strongly emphasized a *whole-person care* approach to their patients, where it was important to know their background and lived experiences when assessing new symptoms and situations. However, a recent study from Norway found that GPs find it difficult to integrate their patients’ stories about difficult life experiences into their clinical work, and their views of the clinical relevance of this varied considerably [36]. This might indicate that our sample of participants had a particular interest in a *whole-person* approach to care, or that they expressed ideal attitudes and perceptions of this approach. However, another recent study of Norwegian GPs showed that they benefitted personally and professionally from a close, trusting relationship with patients such as that was experienced by GPs in the present study in their *familiar territory* with an *extensive relationship* with patients and a *whole-person* approach [37]. This was an important reason to choose and stay in family/general practice over other specialities and the job could, from a theoretical perspective, be seen as a calling [37]. This implies that the underlying idea of *whole-person care* is just as important for GPs themselves, both professionally and privately, as it is for patients. This can explain why *there is a will* from the GPs to help these complex patients and families, and illuminate the frustration they convey when they find that there is *no way* to do so. GPs lose something, affecting both their patients and themselves, when the *unfamiliar territory* results in a fragmented story. The way GPs represent themselves and the CWS in this study with a focus on a fragmented story versus GPs working with whole-person care may also be understood critically and illustrate an often seen tendency to idealize one position against another in cases of potential conflicts and challenging collaborations. Barriers are experienced but also possibly exaggerated as a very usual reaction.

### Unfamiliar territory: challenges to collaboration

In this study the main obstacle to collaboration between GPs and CWS was *unfamiliar territory* regarding CWS’ ways of working, their available tools and how they evaluated cases. This was upheld by a *one-way window of information* and a *closed door to dialogue*. These obstacles were also seen in the data provided by the informants with positive experiences of collaboration with the CWS. However, these GPs had found individual ways to overcome some of these barriers, such as using their personal acquaintance with CWS staff to obtain information in unofficial ways.

With regard to the dimensions in Gittell’s theory of relational coordination, our overall findings indicate low task integration between GPs and CWS [27]. This theory distinguishes three dimensions of relationships (shared goals, shared knowledge and mutual respect) and four dimensions of communication (frequent, timely, accurate and problem-solving communication), which mutually reinforce each other. They form the basis for coordinated collective action. The concepts of *one-way window* and *closed door to dialogue* demonstrate low levels of shared knowledge and challenges in communication. Further, it is difficult to assume mutual respect and shared goals between the parties, in view of the GPs’ narratives and reported consequences of poor collaboration. However, this paper only focuses on data from GPs. The results are therefore not suitable to say anything about the collaboration process per se, as this is a bilateral process in its nature. Further research is needed to look at the collaborative process from both sides.

Our results can be situated in a wider context of interorganizational collaboration, where studies show that one of the important challenges is navigating in unfamiliar organizational contexts [38, 39]. which our study defines as *unfamiliar territory*. Several studies on the collaboration between family/general practice and different health and social care agencies report lack of information sharing as a crucial barrier, as seen in the *one-way window of information* [22, 40]. Studies from Sweden and the USA, focusing directly on CWS and GPs/health services found similar results [16, 18]. The study by Campbell et al. even resulted in a new policy that gave the CWS the possibility to provide a meaningful summary of outcomes and recommendations to health care personnel [18]. Woodmann et al. found that GPs are not, but ought to be, routinely informed when children are referred to social care services, or when families are followed up by other professionals for maltreatment concerns [41]. This is supported by a Danish study finding that a communicative barrier to collaboration is that important information about a patient’s social resources (or lack of these) is not communicated from the social service sector to the health care sector [42]. In order to address this, the *one-way window of information* would need to change to a two-way transfer of useful information. Breimo et al. concluded that it would strengthen trust between the agencies and collaboration if the other party is considered an actual collaborative partner, and not just a source of information and a means to meet a need from the CWS [43]. This means that the *closed door to dialogue* would have to be opened up.

The GPs in our study saw a need for electronic two-way communication with the CWS in order to improve and increase dialogue. A study from Denmark examined collaboration between GPs and the social service sector concerning vulnerable pregnant women. They found similar results and concluded that electronic two-way communication pathways between the GPs and the social service sector would facilitate cross-sectoral communication [42]. Norwegian GPs are used to communicating electronically with hospitals, and primary health and social care services. The CWS offer no possibility for an electronic dialogue that is integrated with the GPs’ electronic patient record system or that maintains a suitable standard of confidentiality. Much of the GPs’ collaborative communication took place after working hours, since they were often too busy during the day to pick up the phone and reach the right people in the CWS. Easy access to two-way communication could increase the dimensions of communication [27]. Communication could be more frequent and more timely, and possibly more accurate and better at problem solving as misunderstandings could be rectified quickly, and joint challenges could be met more effectively, since simple interventions could be implemented sooner. As the two axes of dimensions in Gittell’s theory reinforce each other, this might also enhance the relationship dimensions, developing mutual respect based on shared goals and shared knowledge.

We do not aspire to discuss the legal aspects of the GP’s understanding of confidentiality. However, the perception from the GPs in our study that the CWS adhere to a different level of confidentiality concur with the results of Breimo et al. They found that informants explained the restricted information sharing by the CWS by a particularly strict duty of confidentiality [44].

### Fragmented territory: consequences of collaborative challenges

Our findings also reveal possible consequence of collaborative difficulties for the GPs, namely a feeling of *fragmented territory* that leads to *lost opportunities to help* and *missing pieces* in the patient’s story. Fragmentation of patient pathways as experienced by both patients and health professionals is a topic currently seen as a serious problem for patients’ health in several studies of cross-sectoral treatment [45]. Along these lines, our findings point out that GPs find that collaboration is hampered by factors such as difficult information flow, few opportunities for dialogue and poor knowledge of the partner. Moreover, the experience of fragmentation impedes the ambition of *whole- person care* and the will to collaborate. Although we found few studies that discuss the consequences in this way, it has been shown that information about the outcome of a CWS investigation will affect health care providers’ ability to address the situation in future encounters [18]. Further, information about measures taken because of a social report will indicate the vulnerability of a patient to the GP, and is therefore considered important [42].

### Strengths and limitations

During data collection and analysis, we constantly evaluated the richness and comprehensiveness of the data. We assessed if the data was sufficient to include all relevant nuances in the emerging main themes (constant comparative analysis) and sufficient to ground the theory developed (theoretical saturation). The use of semi-structured individual interviews provided extensive data on the GPs’ experiences. This included informants with both positive and negative experiences of collaboration with the CWS. We consider that our data is saturated in line with the grounded theory approach [46].

According to Malterud, a study with a broad aim, no established theoretical framework (which is the main point of a grounded theory study) and a cross-case analysis strategy will require a larger sample size in order to achieve information power [47]. However, a sample with experience, knowledge and properties specific to the study aim can reach information power with fewer participants. The sampling was purposive, aiming for geographical variation and sufficient experience in family/ general practice. This also ensured that the informants worked in areas where the CWS offices had a variety of organizational structures.

The interviewer had the same professional background as the interviewee, which can strengthen the dialogue and increase the information power, because of professional trust and a shared discourse. This could, however, also lead to preconceptions during the interview, and bias and blind spots in the analysis. To mitigate this, the entire interdisciplinary team was involved in the analysis. MBR was acquainted with one informant but did not participate in the interview situation. OMØ, who did the interviews, had prior acquaintance with four informants. This could skew the data, as the informants might want to please the interviewer or present themselves in a good light. The acquaintance could also mean that the informants felt more comfortable talking about difficult situations. These informants worked in different places of different size, and data from the interviews were in broad terms in accordance with data from informants without prior acquaintance to the authors. To the best of our knowledge we believe that this did not bias the results to such a degree that they are invalid.

## Conclusion

We conclude that GPs perceive collaboration with the CWS as hindered by factors such as difficult information flow, few opportunities for dialogue and limited knowledge of the partner. Despite this, they have a positive attitude towards possible collaboration and an ambition to contribute their professional expertise. Our study is the first to investigate consequences for GPs’ daily practice of difficult collaboration with the CWS. This can possibly lead to lost opportunities for GPs to help families and a lost contribution of GPs’ knowledge and potential actions during a CWS case. It can also lead to fragmentation of patient pathways and of GPs’ knowledge about patients’ history and social situation, which affects their ambition to provide *whole-person care*. We suggest that electronic two-way communication that is effective and satisfies confidentiality standards could make it easier to share information that is relevant for both parties. This could improve the collaborative relationship between GPs and the CWS, lead to better integration of their services, and ultimately benefit vulnerable families.

### Supplementary Information

**Additional file 1. **Consolidated criteria for reporting qualitative studies (COREQ): 32-item checklist

**Additional file 2. **Interview guide.

**Additional file 3. **

## Data Availability

The data set is archived in the UiT Open Research Data Dataverse with restricted access. Anonymized raw data can be available on request by contacting the first author (Oda Martine Steinsdatter Øverhaug, oda.m.overhaug@uit.no).

## Abbreviations

CWS: Child welfare services
GP: General practitioner
