# Supplementary material for: ‘There’s a will, but not a way’: Norwegian GPs’ experiences of collaboration with child welfare services – a grounded theory study
Source: BMC Prim Care. 2024 Jan 24;25:36. doi: 10.1186/s12875-024-02269-9 (PMC10807144; doi:10.1186/s12875-024-02269-9)
Supplement: Supplementary file 2 — Additional file 2. Interview guide. [file 12875_2024_2269_MOESM2_ESM.docx]

Interview guide

- With review date and which interviews the guide was used for.
- Changes highlighted by strikethrough and italics.

| 03.02.2020 | 06.02.2020 | 14.09.2020 | 05.01.2021 |
| --- | --- | --- | --- |
| GP1 | GP2, GP3, GP4, GP5 | GP6, | GP7, GP8, GP9, GP10 |
| There are many children who grow up under difficult conditions, for various reasons. GPs occasionally meet such children, and in many cases the GP has good knowledge of families who are particularly vulnerable. There may be other times where the GP does not know about the difficult situation before child welfare service makes contact, but the GP may still have relevant information. This research project will examine the collaboration between GPs and the child welfare service, and what challenges and opportunities that exists with collaboration.  How long have you worked as a GP?  What is the size of your GP surgery?  Where is the closest child welfare service located?  Introduction   1. Can you start by telling in general terms how it is to be a GP, in situations where you meet vulnerable families and vulnerable children? 2. Can you tell a bit about your collaboration with child welfare service?    - How does it work?    - How do you experience this? 3. Can you tell me about what kind of situations you have contact with child welfare service?   Contact from GP to CWS   1. Can you give an example of a time you contacted child welfare service?    - What happened, how did it go? 2. What do you do when you need to contact child welfare service?    - When/in what situations do you do it?    - How does this work for you and what is you experienced as a GP? 3. Do you have any thoughts about what child welfare service needs from you as a GP?   Notice of concern   1. Can you tell me about sending a notice of concern? 2. Describe what is most challenging about sending such a notice? 3. Can you tell us how you arrive at the decision to send a notice of concern to child welfare service? 4. Can you give examples of how you have collaborated with colleagues, child welfare service or others before sending a notice of concern?    - Why did you collaborate?    - What is your evaluation of such a collaboration? 5. Can you give me an example of when this has been easy and when it has been difficult? 6. Do you have any thoughts on how this process can be made easier for you as a GP?   Contact from CWS to GP   1. Can you tell me about a time when you were contacted by child welfare service?    - What happens?    - How does this work for you and what is you experienced as a GP?    - How does this function as a collaborative tool? 2. What do you think characterizes good and/or bad requests (from CWS)? 3. How do you best convey what you want to convey to child welfare service? 4. Do you have any thoughts on how this could be changed for the better? 5. *Separate sheet with stimulus material* (see below)   Health care for children and follow-up of families   1. Can you tell me about how you follow up on children that are under the care of child welfare service or families who are in contact with child welfare service?    - Do you have any examples of this? 2. How does the follow-up work? 3. What is challenging about these situations? 4. What do you need to provide GP-services to these children and families?   Collaboration between agencies   1. You collaborate with many different agencies, not just child welfare service. What characterizes collaboration that works well for you as a GP? 2. What do you think is necessary in terms of collaboration between GPs and child welfare service to provide help to vulnerable children and families? 3. How do you imagine this collaboration working optimally?   Knowledge of each other as partners   1. What do you think about child welfare services’ knowledge of the work you as GPs do? 2. Is there anything they would have benefited from knowing more about - if so, what? 3. What do you think about your knowledge of the work of child welfare service? 4. What would you like to know more about?   Structural frameworks   1. Which structural framework and conditions in your municipality affect your collaboration with child welfare service? 2. Which structural frameworks and conditions at national level affect your collaboration with child welfare service? 3. Are there other things that can make collaboration difficult? 4. Do you have any thoughts on how this could be changed for the better?   Concluding question   1. Do you have any concluding remarks, or any other comments that we haven’t already touched upon? | There are many children who grow up under difficult conditions, for various reasons. GPs occasionally meet such children, and in many cases the GP has good knowledge of families who are particularly vulnerable. There may be other times where the GP does not know about the difficult situation before child welfare service makes contact.~~, but the GP may still have relevant information.~~ This research project will examine the collaboration between GPs and the child welfare service, and what challenges and opportunities that exists with collaboration. *You are welcome to use examples but remember to anonymize them.*  How long have you worked as a GP?  What is the size of your GP surgery?  Where is the closest child welfare service located?  Introduction   1. Can you start by telling in general terms how it is to be a GP, in situations where you meet vulnerable families and vulnerable children? 2. Can you tell a bit *in general terms* about your collaboration with child welfare service?    - How does it work?    - How do you experience this? 3. Can you *say something general* about what kind of situations you have contact with child welfare service?   Contact from GP to CWS   1. Can you give an example of a time you contacted child welfare service?    - What happened, how did it go?    - *What was the reason you chose to contact child welfare service?*    - *What decide whether you contact them or not?* 2. What do you do when you need to contact child welfare service?    - ~~When/in what situations do you do it?~~    - *At what channels can you contact child welfare service?*    - How does this work for you and what is you experienced as a GP? 3. Do you have any thoughts about what child welfare service needs from you as a GP?   Notice of concern   1. Can you tell me about *the process of* sending a notice of concern? 2. Describe what is most challenging about sending such a notice? 3. Can you tell us how you arrive at the decision to send a notice of concern to child welfare service? 4. Can you give examples of how you have collaborated with colleagues, child welfare service or others before sending a notice of concern?    - Why did you collaborate?    - What is your evaluation of such a collaboration?    - *What is useful with this kind of collaboration?* 5. Can you give me an example of when this has been easy and when it has been difficult? 6. Do you have any thoughts on how this process can be made easier for you as a GP?   Contact from CWS to GP   1. Can you tell me about a time when you were contacted by child welfare service?    - What happens?    - How does this work for you and what is you experienced as a GP?    - How does this function as a collaborative tool? 2. What do you think characterizes good and/or bad requests (from CWS)? 3. How do you best convey what you want to convey to child welfare service? 4. Do you have any thoughts on how this could be changed for the better? 5. *Separate sheet with stimulus material* (see below)   Health care for children and follow-up of families   1. Can you tell me about how you follow up on children that are under the care of child welfare service or families who are in contact with child welfare service?    - Do you have any examples of this? 2. How does the follow-up work? 3. What is challenging about these situations? 4. What do you need to provide GP-services to these children and families?   Collaboration between agencies   1. You collaborate with many different agencies, not just child welfare service. What characterizes collaboration that works well for you as a GP? 2. What do you think is necessary in terms of collaboration between GPs and child welfare service to provide help to vulnerable children and families? 3. How do you imagine this collaboration working optimally?   Knowledge of each other as partners   1. What do you think about child welfare services’ knowledge of the work you as GPs do?    - Is there anything they would have benefited from knowing more about - if so, what? 2. What do you think about your knowledge of the work of child welfare service?    - What would you like to know more about?   Structural frameworks  ~~Which structural framework and conditions in your municipality affect your collaboration with child welfare service?~~  ~~Which structural frameworks and conditions at national level affect your collaboration with child welfare service?~~   1. *Which structural framework and conditions affect your collaboration with child welfare service?*    - *In the municipal?*    - *On a national level?*    - *Economic conditions?*    - *Legal aspects?* 2. Are there other things that can make collaboration difficult? 3. Do you have any thoughts on how this could be changed for the better?   Concluding question   1. Do you have any concluding remarks, or any other comments that we haven’t already touched upon? | There are many children who grow up under difficult conditions, for various reasons. GPs occasionally meet such children, and in many cases the GP has good knowledge of families who are particularly vulnerable. There may be other times where the GP does not know about the difficult situation before child welfare service makes contact. This research project will examine the collaboration between GPs and the child welfare service, and what challenges and opportunities that exists with collaboration. You are welcome to use examples but remember to anonymize them.  How long have you worked as a GP?  What is the size of your GP surgery?  Where is the closest child welfare service located?  Introduction   1. Can you start by telling in general terms how it is to be a GP, in situations where you meet vulnerable families and vulnerable children? 2. Can you tell a bit *in general terms* about your collaboration with child welfare service?    - How does it work?    - How do you experience this? 3. Can you *say something general* about what kind of situations you have contact with child welfare service?   Contact from GP to CWS   1. Can you give an example of a time you contacted child welfare service?    - What happened, how did it go?    - What was the reason you chose to contact child welfare service?    - What decide whether you contact them or not? 2. What do you do when you need to contact child welfare service?    - At what channels can you contact child welfare service?    - How does this work for you and what is you experienced as a GP? 3. Do you have any thoughts about what child welfare service needs from you as a GP?   Notice of concern   1. Can you tell me about the process of sending a notice of concern? 2. Describe what is most challenging about sending such a notice? 3. Can you tell us how you arrive at the decision to send a notice of concern to child welfare service? 4. ~~Can you give examples of how you have collaborated with colleagues, child welfare service or others before sending a notice of concern?~~   *Can you give examples on how you have collaborated with others before you send a notice of concern?*   - - Why did you collaborate?   - What is your evaluation of such a collaboration?   - What is useful with this kind of collaboration?  1. Can you give me an example of when this has been easy and when it has been difficult? 2. Do you have any thoughts on how this process can be made easier for you as a GP?   Contact from CWS to GP   1. Can you tell me about a time when you were contacted by child welfare service?    - What happens?    - How does this work for you and what is you experienced as a GP?    - How does this function as a collaborative tool? 2. What do you think characterizes good and/or bad requests (from CWS)? 3. How do you best convey what you want to convey to child welfare service? 4. Do you have any thoughts on how this could be changed for the better? 5. *Separate sheet with stimulus material* (see below)   Health care for children and follow-up of families   1. Can you tell me about how you follow up on *patients* that are under the care of child welfare service ~~or families who are in contact with child welfare service~~?    - *What about families that are in contact with child welfare service?*    - Do you have any examples of this? 2. How does the follow-up work? *Is it any different than with other patients?* 3. What is challenging about these situations? 4. What do you need to provide GP-services to these children and families?   Collaboration between agencies   1. You collaborate with many different agencies, not just child welfare service. What characterizes collaboration that works well for you as a GP? 2. *Which instruments for collaboration do you find most useful?* 3. *Does GPs and child welfare service need to collaborate?* 4. What do you think is necessary in terms of collaboration between GPs and child welfare service to provide help to vulnerable children and families? 5. How do you imagine this collaboration working optimally?   Knowledge of each other as partners   1. What do you think about child welfare services’ knowledge of the work you as GPs do?    - Is there anything they would have benefited from knowing more about - if so, what? 2. What do you think about your knowledge of the work of child welfare service?    - What would you like to know more about?   Structural frameworks   1. Which structural framework and conditions affect your collaboration with child welfare service?    - In the municipal?    - On a national level?    - Economic conditions?    - Legal aspects? 2. Are there other things that can make collaboration difficult? 3. Do you have any thoughts on how this could be changed for the better?   Concluding question   1. Do you have any concluding remarks, or any other comments that we haven’t already touched upon? | There are many children who grow up under difficult conditions, for various reasons. GPs occasionally meet such children, and in many cases the GP has good knowledge of families who are particularly vulnerable. There may be other times where the GP does not know about the difficult situation before child welfare service makes contact. This research project will examine the collaboration between GPs and the child welfare service, and what challenges and opportunities that exists with collaboration. You are welcome to use examples but remember to anonymize them.  How long have you worked as a GP?  What is the size of your GP surgery?  Where is the closest child welfare service located?  Introduction   1. Can you start by telling in general terms how it is to be a GP, in situations where you meet vulnerable families and vulnerable children? 2. Can you tell a bit in general terms about your collaboration with child welfare service?    - How does it work?    - How do you experience this? 3. Can you say something general about what kind of situations you have contact with child welfare service?   Contact from GP to CWS   1. Can you give an example of a time you contacted child welfare service?    - What happened, how did it go?    - What was the reason you chose to contact child welfare service?    - What decide whether you contact them or not? 2. What do you do when you need to contact child welfare service?    - At what channels can you contact child welfare service?    - How does this work for you and what is you experienced as a GP? 3. Do you have any thoughts about what child welfare service needs from you as a GP? 4. *What are your options for dialogue with child welfare service? Do you have any examples?*   Notice of concern   1. Can you tell me about the process of sending a notice of concern? 2. Describe what is most challenging about sending such a notice? 3. Can you tell us how you arrive at the decision to send a notice of concern to child welfare service? 4. *What are your thoughts concerning cases where you are in doubt about sending a notice of concern, in contrast to cases of more clinical character where you are in doubt about what to do?* 5. *What possibilities do you have to learn from, or to get better at handling, cases that involves child welfare service?* 6. Can you give examples on how you have collaborated with others before you send a notice of concern?    - Why did you collaborate?    - What is your evaluation of such a collaboration?    - What is useful with this kind of collaboration? 7. Can you give me an example of when this has been easy and when it has been difficult? 8. Do you have any thoughts on how this process can be made easier for you as a GP?   Contact from CWS to GP   1. Can you tell me about a time when you were contacted by child welfare service?    - What happens?    - How does this work for you and what is you experienced as a GP?    - How does this function as a collaborative tool? 2. What do you think characterizes good and/or bad requests (from CWS)? 3. How do you best convey what you want to convey to child welfare service? 4. Do you have any thoughts on how this could be changed for the better? 5. *Separate sheet with stimulus material* (see below)   Health care for children and follow-up of families   1. Can you tell me about how you follow up on patients that are under the care of child welfare service?    - ~~What about families that are in contact with child welfare service?~~    - Do you have any examples of this?    - How does this follow-up work? Is it any different than with other patients?    - What is challenging about these situations? 2. *Do you have experience with follow-up on families or children that have contact with child welfare service - tell me about it?*    - What do you need to provide GP-services to these children and families? 3. *Will the involvement of child welfare service affect your work with your patient? How? Why?*    - *Do you have any examples?*    - *Do you think that information about an earlier or ongoing child welfare-case is relevant for you, concerning your follow up on these patients? If yes, why?* 4. *Some informants have expressed that they don’t feel appreciated in the follow up of these children or families What are your thoughts on that? What are your experiences?*   Collaboration between agencies   1. You collaborate with many different agencies, not just child welfare service. What characterizes collaboration that works well for you as a GP? 2. Which instruments for collaboration do you find most useful? 3. Does GPs and child welfare service need to collaborate?  - What do you think is necessary in terms of collaboration between GPs and child welfare service to provide help to vulnerable children and families?  1. How do you imagine this collaboration working optimally? 2. *How do you imagine this collaboration working optimally concerning patients struggling with drug addiction or mental illness, that are also being followed up by child welfare service? (E.g. when doing drug tests?)*   Knowledge of each other as partners   1. What do you think about child welfare services’ knowledge of the work you as GPs do?    - Is there anything they would have benefited from knowing more about - if so, what? 2. What do you think about your knowledge of the work of child welfare service?    - What would you like to know more about?   Structural frameworks   1. Which structural framework and conditions affect your collaboration with child welfare service?    - In the municipal?    - On a national level?    - Economic conditions?    - Legal aspects? 2. Are there other things that can make collaboration difficult? 3. Do you have any thoughts on how this could be changed for the better?   Concluding question   1. Do you have any concluding remarks, or any other comments that we haven’t already touched upon? |

**Stimulus material/fact sheet, used in question 17.** The fact sheet shows different types of content of concern in cases presented to child welfare service. The analysis of relationships between referral content and request for information from the GP (see question 17 below) was conducted specifically for this study and is not previously published. During the interview the fact sheet was shown to the informant, on paper or on the screen (for the online interviews). Question 17 was read by the interviewer, and contents with red markings were pointed out specifically during the question formulation.

The figure in the fact sheet was published by Vis, et. al in Norwegian in 2020, Barnevernets undersøkelsesarbeid. Samlede resultater og anbefalinger. (‘Child welfare services investigation and assessment of reported cases', Regional Centre for Child and Youth Mental Health & Child Welfare, UiT The Arctic University of Norway, Tromsø, Norway)

The figures in brackets are % of all cases.


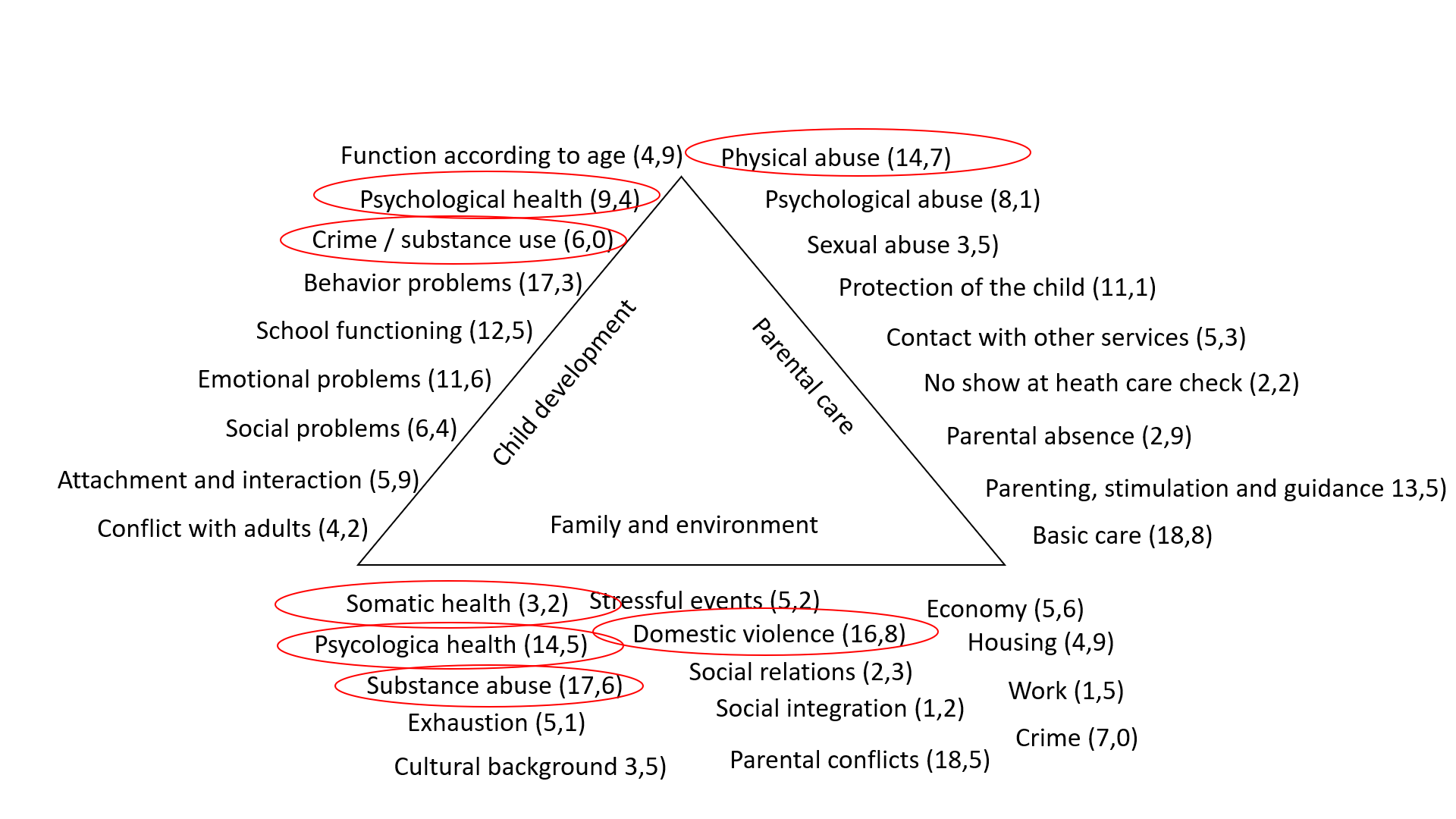


Question 17/20:

The type of concern child welfare service has can determine whether they request information from the GP or not. In total, information is obtained from the GP in 30.8% of all child welfare cases. In cases where there is concern about the child's crime/ substance use and psychological health, information is requested from the GP in between 10 and 20% of the cases. In cases where there is concern about the parents' drug use, mental or somatic health, domestic violence and physical abuse of the child, information is requested from the GP in between 30 and 40% of the cases.

What do you think about these numbers?

In which areas do you think that you as a GP can contribute with information?
