# Supplementary material for: ‘There’s a will, but not a way’: Norwegian GPs’ experiences of collaboration with child welfare services – a grounded theory study
Source: BMC Prim Care. 2024 Jan 24;25:36. doi: 10.1186/s12875-024-02269-9 (PMC10807144; doi:10.1186/s12875-024-02269-9)
Supplement: Supplementary file 3 — Additional file 3. [file 12875_2024_2269_MOESM3_ESM.pdf]

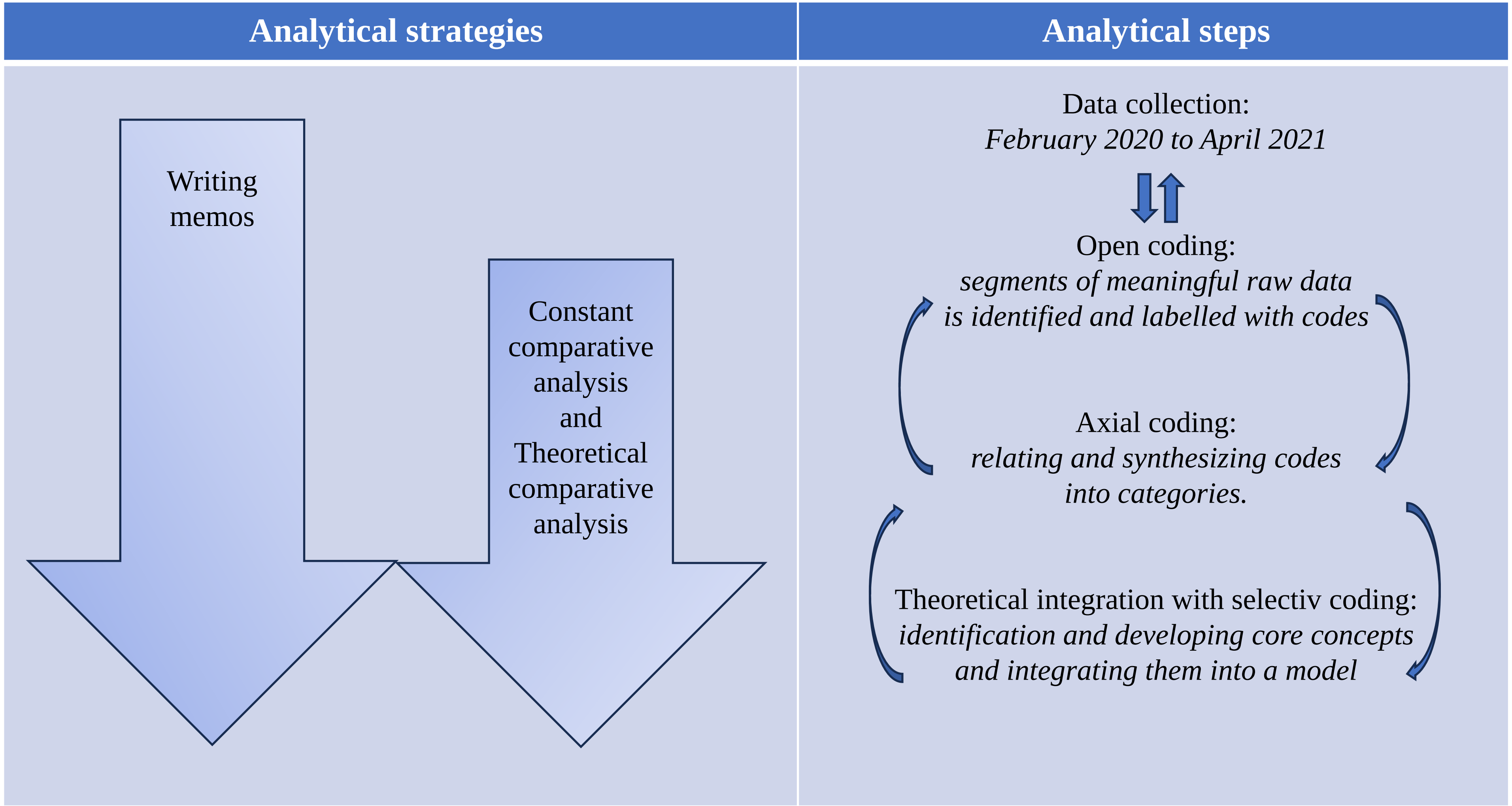
Analytical strategies
Analytical steps
Writing memos
Constant comparative analysis and Theoretical comparative analysis
Data collection:
February 2020 to April 2021
Open coding:
segments of meaningful raw data is identified and labelled with codes
Axial coding:
relating and synthesizing codes into categories.
Theoretical integration with selectiv coding:
identification and developing core concepts and integrating them into a model

Extract from GP1	Open coding	Axial coding	Selective coding, development of concept
It's not a collaboration - it's a request. I don't think we are allowed to withhold information.	Requests are not collaboration<br>GPs are obliged to give information	One way stream of information	One way window:<br>Between the GP and the CWS there is a one way window, related to the information flow. The GPs don't see their own obligation of confidentiality as a piece in this hindrance, but they experience that the CWS have a stronger obligation of confidentiality than themselves. The GPs feel that they have to give everything but get nothing back. This one way window of information is perceived as a huge hinder for collaboration.<br><br>This one way window makes the GPs concerned about the information they are requested to send about their patients. They don't know what legal basis the CPS have for the information request, as they don't get any information about the case, and some GPs experience that CWS requests information they can't see the relevance of. (…)
**Extract from GP3**			
I think sometimes that the law should be changed, so the CPS have an opening to tell a bit more to the GP about why they need information.			
**Extract from GP4**			
where there is a dialogue - that's what characterizes collaboration. . (…)	Collaboration is chatacterized by dialogue	Collaboration requires dialogue	
And to a certain extent we have that with NAV (the labour and welfare administration) - yes, to a certain extent anyway.			
There is at least an exchange of messages there - we may not always agree, but there is now at least a - it is at least open for dialogue.	Possibility of dialogue back and forth		
But with child protection, I feel there is only a one way window.	With CWS there is only a one way mirror	One way window	
**Extract from GP8**			
Eh, I think that - so - the fact that they keep their cards so close to their chest, heh, eh - I think it's a structural framework that - which is a bit negative for cooperation.	Little information is negative for the collaboration	One way window	
eh, at the same time - the more I get to know them the easier it is to pick up the phone and yes, get some information anyway, without it having to be so official.	Personal knowledge is important to get around the obstacle with little information	Holes in the one way window	
